# Low Prevalence of Hepatitis B and C Virus Markers among Children and Adolescents

**DOI:** 10.1155/2014/324638

**Published:** 2014-07-01

**Authors:** Livia Melo Villar, Luciane Almeida Amado, Adilson José de Almeida, Vanessa Salete de Paula, Lia Laura Lewis-Ximenez, Elisabeth Lampe

**Affiliations:** ^1^Laboratory of Viral Hepatitis, Oswaldo Cruz Institute, FIOCRUZ, 21040360 Rio de Janeiro, RJ, Brazil; ^2^Laboratory of Technological Development of Virology, Oswaldo Cruz Institute, FIOCRUZ, 21040360 Rio de Janeiro, RJ, Brazil

## Abstract

This study aimed to determine the prevalence of HBV and HCV among children and adolescents attending schools and daycare centres in Rio de Janeiro State, located in southern Brazil. Serum samples from 1,217 individuals aged 0 to 18 years were collected from 1999 to 2012 and tested for HBsAg, total anti-HBc, anti-HBs, and anti-HCV by ELISA. Reactive HBsAg and anti-HBc samples were tested for HBV DNA. Reactive anti-HCV samples were tested for HCV RNA and genotyped by RFLP. HBsAg was detected in 1.8% of individuals, and total anti-HBc was detected among 3.6% of individuals. Anti-HBs reactivity was found among 25.3% (322/1,217) of the individuals and increased from 6.28% in the years 1999-2000 to 76.2% in the years 2001–2012 (*P* < 0.0001). HBV DNA was detected in 18 of 51 individuals who presented with HBsAg or isolated anti-HBc, and nine were considered occult hepatitis B cases. Three individuals were anti-HCV- and HCV RNA-positive: two of them were infected with genotype 1, and the other was infected with genotype 3. Low levels of HBV and HCV markers were observed in children and adolescents. HBV immunity increased during the period of study, indicating that childhood universal HBV vaccination has been effective for controlling HBV infection in Brazil.

## 1. Introduction 

Hepatitis B virus (HBV) and hepatitis C virus (HCV) infections are important public health problems with broad clinical spectrums, from asymptomatic infection to cirrhosis and hepatocellular carcinoma [1, 2]. According to the World Health Organisation (WHO), approximately 240 million people are chronically infected with HBV worldwide, while 150 million people are infected with HCV [2, 3].

Recently, an epidemiological survey for HBV and HCV infection was conducted among individuals aged 10 to 69 years living in the five geographic regions of Brazil, and this survey reported the overall HBsAg, anti-HBc, and anti-HCV seroprevalence rates of 0.37%, 7.4%, and 1.38%, respectively [4]. Among individuals aged 10 to 19 years, the prevalence of anti-HCV was 0.75% [5], and 1.1% of the individuals were anti-HBc reactive [4]. Most of the prevalence studies for HBV and HCV infections were conducted among blood donors or specific groups, such as drug users [6, 7]. In Brazil, a few studies regarding the seroprevalence of HBV and HCV markers were conducted among children and the adolescent population; these studies reported prevalences ranging from 0 to 0.7% for HBsAg, 0.5 to 1.4% for anti-HBc, 48.6 to 58.8% for anti-HBs, and 0% for anti-HCV [8–10].

The availability of safe and efficacious vaccines has led to the feasibility of effective control of HBV infection, especially in areas of high prevalence where most chronic HBV carriers acquire the infection very early in life [11]. In Brazil, HBV vaccination became mandatory for all newborns in 1997, and in 2001, the National Immunization Program was extended to the population of individuals up to 19 years old [12]. Therefore, most children born before 1997 could not be protected against HBV infection, and these individuals could become chronic carriers of the virus.

Hepatitis B and C share common transmission pathways; thus, it is possible to investigate them simultaneously [2, 3]. The prevalence of HBV and HCV markers in children varies by risk factors and geographic location [3, 13, 14]. Children from all parts of the world who received multiple blood transfusions before 1992 have a 50% to 95% chance of being HCV-positive [15]. Moreover, it is well known that adolescents are exposed to increased risk factors, such as unprotected sexual relations, tattooing, and body piercing, which can lead to HBV and/or HCV infection [16]. Thus, a serological survey was performed among children and adolescents from Rio de Janeiro State, located in southern Brazil, to evaluate the changes in HBV and HCV marker profiles according to age group.

## 2. Materials and Methods

### 2.1. Study Design

This was a retrospective study that aims to evaluate the prevalence of serological markers for HBV and HCV infections among children and adolescents from a metropolitan region of Rio de Janeiro State.

### 2.2. Studied Population

In the present study, daycare centres and schools from a metropolitan region of Rio de Janeiro State were analysed. Rio de Janeiro State is the third most populous state in Brazil and is divided into six regions (Lowland, Centre, Metropolitan, Northeast, North, and South). Approximately 80% of the individuals of the state live in a metropolitan region, and 40% of them were aged 0 to 19 years and attended daycare centres or schools in 2010 [17]. The study population was from a metropolitan region of Rio de Janeiro that corresponds to an urban area of the state.

The sample included all the children attending four primary schools and two daycare centres located in the metropolitan region of Rio de Janeiro State between 1999 and 2012. All individuals invited to participate in the study were included in this study. Schools were selected using a nonprobability sampling method, and only public schools and daycare centres located in the metropolitan region of Rio de Janeiro were included. The schools included both primary and secondary schools, while daycare centres included kindergartens and prekindergartens.

### 2.3. Data Collection and Processing

The inclusion criteria were as follows: age between 0 to 18 years and presentation of an informed consent form signed by parents or other persons who were legally responsible for the participants. Trained community health workers (CHWs) explained the objectives of the study to the participants, who were then asked to obtain written informed consent from their parents before blood sample collection. This study was approved by the Ethics Committee in Human Research at the Oswaldo Cruz Foundation.

Serum samples were tested for HBsAg, total anti-HBc, anti-HBs, and anti-HCV using commercial immunoassays supplied by Diasorin (Italy). HBsAg and isolated anti-HBc reactive samples were submitted to seminested PCR testing for HBV DNA detection [18] to identify active HBV infection. Anti-HCV reactive samples were submitted to nested PCR testing, and the samples that contained HCV RNA were also genotyped by RFLP [19, 20]. The molecular methods had sensitivity of 50 UI/mL [19], and all serum samples were stored at −20°C until analysis.

All individuals who tested positive for anti-HCV/HCV RNA and HBsAg were referred to hepatology units in the public health system, where these individuals would receive the appropriate antiviral treatment. Information regarding the type of treatment was not available for this study.

### 2.4. Statistical Analysis

The prevalence rates of HBV and HCV markers were calculated for the total studied population and in relation to gender, age group, institution groups, and period of HBV vaccination available at public health units for children and adolescents as of 2001. Continuous variables were reported as the mean ± standard deviation. HBsAg, anti-HBc, anti-HBs, and anti-HCV were analysed by comparing the children according to the following age groups: infant (1 month to 2 years old), pre-school age (3 to 6 years old), school age (7 to 11 years old), adolescents (12 to 15 years old), and young individuals (16 to 18 years old).

The differences in HBsAg, anti-HBc, anti-HBs, and anti-HCV prevalence rates among these various groups were tested for statistical significance by the Chi-square for trend test using GraphPad Instat, version 3.06. The results were considered statistically significant when *P* < 0.05.

## 3. Results

A total of 1,217 children and adolescents attending daycare centres and schools from Rio de Janeiro were included in this study. The mean age was 10.39 ± 4.11 years, ranging from 0 to 18 years with 51.6% females.  Table 1 shows the HBV and HCV markers among the population studied according to gender, age group, and period of HBV vaccination available at public health units. In the total group, previous HBV infection (HBsAg−/anti-HBc+) was observed in 3.6% (44/1,217) of individuals. A total of 1.2% of individuals (15/1,217) also had anti-HBs markers, indicating HBV immunity, and 2.4% had isolated anti-HBc.

Active HBV infection (HBsAg+) was observed in 22 individuals (1.8%), most of whom were males (*n* = 14) aged 7 to 11 years old (*n* = 9). Most of the individuals did not have any HBV markers (70.3%), especially those aged 12 to 15 years (44.9%).

HBV immunity (as determined by anti-HBs positivity) was observed in 25.3% (308/1,217) of individuals. A total of 24.1% of these individuals were most likely vaccinated because only anti-HBs was detected in their serum samples.

When HBV immunity, previous HBV infection, and HBV active infection were evaluated according to the HBV vaccination records available at public health units in Brazil, it was observed that anti-HBs reactivity increased from 6.28% during 1999-2000 (55/885) to 76.2% (253/332) during 2001–2012 (*P* < 0.0001). On the other hand, HBsAg and anti-HBc reactivity decreased from 2.2% for HBsAg (20/885) and 4.6% (41/885) for anti-HBc during 1999-2000 to 0.6% (2/332) for HBsAg and 2.1% (7/332) for anti-HBc during 2001–2012. These results were statistically significant for anti-HBc markers (*P* = 0.003) according to the year of sample collection.

Positivity for HBsAg and anti-HBc was not statistically significant with respect to gender and age group. Anti-HBs positivity was associated with age group, as most individuals aged between 12 and 15 years had HBV immunity (103/308) (*P* < 0.0001).

Among the 51 individuals who had HBsAg (*n* = 22) or isolated anti-HBc (*n* = 29), 18 were also positive for HBV DNA, nine had active infections (HBsAg-reactive sample), and the other 9 individuals were considered occult hepatitis B cases (HBsAg−, HBV DNA+, anti-HBc+, and anti-HBs−).

Regarding HCV infection, 20 individuals had anti-HCV antibodies in serum samples, but only 3/20 individuals were HCV RNA-positive. The HCV patients were mostly males (2/3), and their ages were 7, 11, and 18 years. Two of them were infected with genotype 1, and the other patient was infected with genotype 3.

## 4. Discussion

The present study was conducted to evaluate the seroprevalence of HBV and HCV markers among children and adolescents from Rio de Janeiro State. Low frequencies of HBV and HCV infections were observed. HBsAg prevalence decreased during the study period, with prevalence varying from 2.2% between 1999 and 2000 to 0.6% between 2001 and 2012. HBsAg prevalence from 2001 to 2012 was similar to previous studies among children and adolescents from the southern region of Brazil conducted between 2007 and 2008 (the prevalence varied from 0.2 to 0.7%) [8, 9]. On the other hand, the HBsAg prevalence found in the present study was lower than that observed among children from Indonesia (3.1%) [21], Mexico (3.1%) [22], and North India (4.35%) [13], independent of the year of sample collection. When considering the detection of HBsAg and/or HBV DNA, active HBV infection was observed in 31/1,217 (2.5%) individuals.

Among children and adolescents from Rio de Janeiro State, 4.6% were anti-HBc-positive from 1999 to 2000, and 2.1% had this marker from 2001 to 2012. From 2001 to 2012, the prevalence of anti-HBc was lower than that described in studies of children and adolescents from the southern region of Brazil conducted between 2007 and 2008 (ranging from 1 to 1.4%) [8, 9] and among hospitalised children in Mexico (1%) [22].

Positivity for HBsAg and anti-HBc was not associated with age group; however, high prevalence was observed among older age groups. A similar result was observed among individuals from other geographical areas of Brazil [4]. On the other hand, anti-HBs positivity was associated with age group, as most individuals aged between 12 and 15 years had HBV immunity. This result is similar to observations among children aged 10 to 14 years in China [23].

HBV DNA was also detected among 18 individuals. Of these individuals, nine had an active infection (HBsAg reactive sample), while the other 9 individuals were thought to have occult hepatitis B infection (reactivity to anti-HBc alone). In the present study, a higher frequency (31%, 9/29) of suggested cases of occult hepatitis B infection was observed when compared to the frequency of HBV-infected children with haematological disorders in Egypt (20%) [24].

Regarding HBV immunity, a relatively lower level of anti-HBs seropositivity (25.3%) was found in the population during the entire period of study when compared to other similar studies conducted in southern Brazil (48.6%) [8] and Iran (60%) [25]. However, a higher anti-HBs prevalence rate was observed after 2001, most likely due to implementation of HBV vaccination in public health units. Although information regarding the previous history of HBV vaccination of children was not available at the time of sample collection, higher rates of anti-HBs prevalence after 2001 suggest that universal HBV vaccination has improved HBV immunity in this population as it has in China [26].

The successful introduction of the HBV vaccine into the Brazilian National Immunization Program has had a great impact on the prevalence of HBV markers among children. The results of the present study showed that universal vaccination of infants has contributed directly to the reduction in the prevalence of HBsAg and anti-HBc during the years of sample collection. These data are consistent with studies conducted in other parts of the world [11, 23, 25, 26], which have shown a reduction in HBV marker prevalence since the introduction of the hepatitis B vaccination programs.

Regarding HCV infection, 20 individuals were anti-HCV reactive; however, the infection was confirmed in only three cases by HCV RNA detection, showing a similar HCV prevalence (0.2%) when compared to one study conducted among healthy children from Taiwan (0.3%) in which no children had HCV viremia [27]. In Brazil, no HCV cases were reported in studies with children aged 10 to 16 years from southern Brazil [8–10], although low HCV prevalence was observed in individuals aged 10 to 19 years from different geographical regions of Brazil (0.75%) [5]. These data appear to show that differences in HCV prevalence could be observed according to the group of children and the geographical area evaluated. Regarding HCV genotypes, the same global distribution was observed, as most of the samples belonged to genotype 1 (75%), which is the most common HCV genotype worldwide and among HCV infected individuals from Brazil [3, 5, 28].

## 5. Conclusions

In conclusion, low prevalence of HBV and HCV infections was observed in children and adolescents during the study period. In addition, HBV immunity increased among these individuals during the study period, indicating that childhood universal HBV vaccination has been effective for HBV control in Brazil. Furthermore, HCV prevalence was considered low in this population. These results highlight the importance of prevention programs for children and adolescents, both to avoid risk behaviour and to maintain the low prevalence of hepatitis B and C among these groups of individuals.

## Figures and Tables

**Table 1:** Hepatitis B and C virus markers among children and adolescents attending six institutions in Rio de Janeiro State (*n* = 1,217).

	Number tested	HBsAg− anti-HBc+ anti-HBs+	HBsAg− anti-HBc+ anti-HBs−	HBsAg− anti-HBc− anti-HBs+	HBsAg+ anti-HBc− anti-HBs−	HBsAg+ anti-HBc+ anti-HBs−	HBsAg− anti-HBc− anti-HBs−	anti-HCV+ HCV RNA+
Total population studied	**1217 (100%)**	**15 (1.2%)**	**29 (2.4%)**	**293 (24.1%)**	**18 (1.5%)**	**4 (0.3%)**	**855 (70.3%)**	**3 (0.2%)**
Sex								
Female	628 (51.6%)	7 (46.7%)	16 (55.2%)	161 (54.9%)	6 (33.3%)	2 (50%)	434 (50.8%)	1 (33.3%)
Male	589 (48.4%)	8 (53.3%)	13 (44.8%)	132 (45.1%)	12 (66.6%)	2 (50%)	421 (49.2%)	2 (66.6%)
Age group (years)								
0–2	36 (2.9%)	1 (6.7%)	0 (0%)	24 (8.2%)	1 (5.5%)	1 (25%)	9 (1.0%)	0 (0.0%)
3–6	221 (18.2%)	1 (6.7%)	3 (10.3%)	87 (29.7%)	2 (11.1%)	1 (25%)	126 (14.7%)	0 (0.0%)
7–11	369 (30.3%)	7 (46.6%)	8 (27.6%)	53 (18.1%)	8 (44.4%)	1 (25%)	290 (33.9%)	2 (66.6%)
12–15	513 (42.2%)	6 (40%)	18 (62.1%)	97 (33.1%)	7 (38.9%)	1 (25%)	384 (44.9%)	0 (0.0%)
16–18	78 (6.4%)	0 (0.0%)	0 (0.0%)	32 (10.9%)	0 (0.0%)	0 (0.0%)	46 (5.4%)	1 (33.3%)
Period of HBV vaccination available at public health units∗								
Group 1	885 (72.7%)	11 (73.3%)	28 (96.5%)	44 (15.0%)	18 (100.0%)	2 (50.0%)	782 (91.5%)	3 (100.0%)
Group 2	332 (27.3%)	4 (26.7%)	1 (3.5%)	249 (85.0%)	0 (0.0%)	2 (50.0%)	73 (8.5%)	0 (0.0%)

*HBV vaccination available at public health units before 2001 (Group 1) and HBV vaccination available at public health units as of 2001 (Group 2).

## References

[1] Tan Y-J (2011). Hepatitis B virus infection and the risk of hepatocellular carcinoma. *World Journal of Gastroenterology*.

[2] World Health Organization Hepatitis C. http://www.who.int/mediacentre/factsheets/fs164/en/.

[3] World Health Organization Hepatitis B. http://www.who.int/mediacentre/factsheets/fs204/en/.

[4] Pereira LMMB, Martelli CMT, Merchán-Hamann E (2009). Population-based multicentric survey of hepatitis B infection and risk factor differences among three regions in Brazil. *American Journal of Tropical Medicine and Hygiene*.

[5] Pereira LMMB, Martelli CMT, Moreira RC (2013). Prevalence and risk factors of Hepatitis C virus infection in Brazil, 2005 through 2009: a cross-sectional study. *BMC Infectious Diseases*.

[6] Nascimento MC, Mayaud P, Sabino EC, Torres KL, Franceschi S (2008). Prevalence of hepatitis B and C serological markers among first-time blood donors in Brazil: a multi-center serosurvey. *Journal of Medical Virology*.

[7] Nelson PK, Mathers BM, Cowie B (2011). Global epidemiology of hepatitis B and hepatitis C in people who inject drugs: results of systematic reviews. *The Lancet*.

[8] do Livramento A, de Cordova CMM, Spada C, Treitinger A (2011). Seroprevalence of hepatitis B and C infection markers among children and adolescents in the Southern Brazilian region. *Revista do Instituto de Medicina Tropical de Sao Paulo*.

[9] Scaraveli NG, Passos AM, Voigt AR (2011). Seroprevalence of hepatitis b and hepatitis C markers in adolescents in Southern Brazil. *Cadernos de Saude Publica*.

[10] Voigt AR, Neto MS, Spada C, Treitinger A (2010). Seroprevalence of hepatitis b and hepatitis C markers among children and adolescents in the south brazilian region—metropolitan area of Florianópolis, Santa Catarina. *Brazilian Journal of Infectious Diseases*.

[11] Luo Z, Li L, Ruan B (2012). Impact of the implementation of a vaccination strategy on hepatitis B virus infections in China over a 20-year period. *International Journal of Infectious Diseases*.

[12] Ministry of Health, Brazil Instructional material for training in surveillance of viral hepatitis. http://www.aids.gov.br/sites/default/files/cbve_hepatites.pdf.

[13] Qamer S, Shahab T, Alam S, Malik A, Afzal K (2004). Age-specific prevalence of hepatitis B surface antigen in pediatric population of Aligarh, North India. *Indian Journal of Pediatrics*.

[14] Porto AF, Tormey L, Lim JK (2012). Management of chronic hepatitis C infection in children. *Current Opinion in Pediatrics*.

[15] Jonas MM (2002). Children with hepatitis C. *Hepatology*.

[16] Meheus A (2000). Teenagers’ lifestyle and the risk of exposure to hepatitis B virus. *Vaccine*.

[17] Brazilian Institute of Geography and Statistics 2010 Census. http://www.ibge.gov.br/estadosat/perfil.php?sigla=rj.

[18] Portilho MM, Martins PP, Lampe E, Villar LM (2012). A comparison of molecular methods for hepatitis B virus (HBV) DNA detection from oral fluid samples. *Journal of Medical Microbiology*.

[19] de Almeida AJ, Campos-de-Magalhães M, Brandão-Mello CE, de Oliveira RV, Yoshida CF, Lampe E (2009). Detection of hepatitis C virus in platelets: evaluating its relationship to viral and host factors. *Hepatogastroenterology*.

[20] Oliveira MLA, Bastos FI, Sabino RR (1999). Distribution of HCV genotypes among different exposure categories in Brazil. *Brazilian Journal of Medical and Biological Research*.

[21] Utsumi T, Yano Y, Lusida MI, Amin M, Hotta H, Hayashi Y (2010). Serologic and molecular characteristics of hepatitis B virus among school children in East Java, Indonesia. *American Journal of Tropical Medicine and Hygiene*.

[22] Escobedo-Meléndez G, Fierro NA, Roman S, Maldonado-González M, Zepeda-Carrillo E, Panduro A (2012). Prevalence of hepatitis A, B and C serological markers in children from western Mexico. *Annals of Hepatology*.

[23] Xiao J, Zhang J, Wu C (2012). Impact of hepatitis B vaccination among children in Guangdong Province, China. *International Journal of Infectious Diseases*.

[24] Said ZNA, El-Sayed MH, El-Bishbishi IA (2009). High prevalence of occult hepatitis B in hepatitis C-infected Egyptian children with haematological disorders and malignancies. *Liver International*.

[25] Aghakhani A, Banifazl M, Izadi N (2011). Persistence of antibody to hepatitis B surface antigen among vaccinated children in a low hepatitis B virus endemic area. *World Journal of Pediatrics*.

[26] Shen L, Wang F, Wang F (2012). Efficacy of yeast-derived recombinant hepatitis B vaccine after being used for 12 years in highly endemic areas in China. *Vaccine*.

[27] Wu T-C, Chuang W-L, Dai C-Y (2008). Hepatitis C virus infection among children in aboriginal areas in Taiwan. *Transactions of the Royal Society of Tropical Medicine and Hygiene*.

[28] Paraboni MLR, Sbeghen MD, Wolff FH, Moreira LB (2012). Risk factors for infection with different hepatitis C virus genotypes in southern Brazil. *The Scientific World Journal*.

